# Severity-stratified genetic diagnosis by trio exome sequencing in isolated fetal growth restriction

**DOI:** 10.3389/fgene.2026.1774321

**Published:** 2026-02-26

**Authors:** Ying Li, Weiheng Deng, Minghui Meng, Xiaomin Lu, Mei Guan, Haitang Wei, Xigui Long, Ting Qin

**Affiliations:** 1 Medical Genetics and Prenatal Diagnosis Center, Guangxi Academy of Medical Sciences and the People’s Hospital of Guangxi Zhuang Autonomous Region, Nanning, China; 2 Department of Obstetrics, Guangxi Academy of Medical Sciences and the People’s Hospital of Guangxi Zhuang Autonomous Region, Nanning, China

**Keywords:** copy number variation sequencing, exome sequencing, fetal growth restriction, genetic syndrome, prenatal diagnosis, preeclampsia

## Abstract

**Background:**

Exome sequencing (ES) is increasingly used in prenatal diagnosis. However, its efficacy for isolated fetal growth restriction (FGR), especially across different levels of severity, is not well established. This study sought to evaluate and compare the diagnostic yield and clinical impact of trio-ES between isolated non-severe and severe FGR cases.

**Methods:**

In this retrospective study, 164 singleton pregnancies with isolated FGR were stratified into non-severe FGR (estimated fetal weight [EFW] between the third and 10th percentiles) and severe FGR (EFW <third percentile). All cases underwent chromosomal karyotyping and copy number variation sequencing. In parallel, trio-ES was performed in 125 cases. The diagnostic yield of trio-ES was then compared between the severity groups.

**Results:**

Pathogenic or likely pathogenic (P/LP) variants were identified via trio-ES in 8.3% (3/36) of non-severe FGR cases and 18.0% (16/89) of severe FGR cases. In the non-severe group, all detected P/LP variants were associated with high-risk phenotypes and led to termination of pregnancy. In the severe group, these variants were associated with moderate-to-severe disorders, and pregnancy outcomes were diverse (50% termination). Furthermore, the severe FGR cohort exhibited a higher prevalence of preeclampsia and abnormal umbilical artery Doppler waveforms compared to the non-severe group, with adverse outcomes attributable to both monogenic disorders and maternal-placental factors.

**Conclusion:**

This stratified analysis demonstrates that ES provides substantial diagnostic value across the entire severity spectrum of isolated FGR, identifying clinically significant monogenic disorders in both severe and non-severe cases. These findings support the inclusion of ES in the diagnostic workup of isolated FGR, regardless of strict severity cut-offs. They also highlight the need for integrated genetic counseling to manage variants of uncertain significance and multifactorial risks, particularly in severe cases.

## 1 Introduction

Fetal growth restriction (FGR) is a condition in which a fetus fails to achieve its expected biological growth potential. It is associated with increased risks of adverse perinatal outcomes and may have long-term consequences for neurodevelopment and metabolic health. The etiology of FGR is multifactorial, involving maternal, placental, fetal, and environmental determinants (Nowakowska et al., 2021). Among fetal factors, genetic anomalies constitute a significant cause, especially in cases of early onset or severe growth restriction. The reported prevalence of genetic abnormalities in FGR cohorts ranges from 3.9% to 19%. This variability primarily stems from differences in the diagnostic thresholds applied (e.g., specific estimated fetal weight [EFW] percentile cutoffs), the genetic testing techniques used, and whether FGR is isolated or associated with other structural anomalies (Snijders et al., 1993; Wu et al., 2023).

A study demonstrates that a combined approach using karyotyping and chromosomal microarray analysis (CMA) detects pathogenic or likely pathogenic (P/LP) copy number variants (CNVs) in approximately 12.4% of FGR cases. The diagnostic yield of karyotyping alone is about 6.7%, while CMA independently establishes a diagnosis in approximately 11.9% of cases (Cai et al., 2022). Supporting this, another study reported that CMA provides an incremental diagnostic yield of 10% over karyotyping in FGR cohorts, with a 4% specific gain in isolated FGR cases (Borrell et al., 2018). However, despite these advances, conventional cytogenetic and molecular genetic testing fails to establish a definitive etiological diagnosis in a significant proportion of FGR cases, particularly those classified as isolated.

Exome sequencing (ES) has emerged as a powerful tool in prenatal genetic diagnosis. Although current professional guidelines do not specifically address its use in isolated FGR, emerging evidence suggests that ES can provide an additional diagnostic yield of approximately 12% for isolated FGR cases with normal karyotype and CMA results (Pauta et al., 2023). Nevertheless, significant heterogeneity exists among studies evaluating trio-ES in isolated FGR, mainly due to differences in inclusion criteria. One study using an EFW below the 10th percentile reported a diagnostic yield of 36.36% (Chen et al., 2025), whereas another applying a stricter cutoff (EFW <third percentile) found a yield of 15.7% (Zhou et al., 2023). There is a limited number of studies systematically comparing the diagnostic efficacy of ES across different strata of FGR severity.

Applying fetal growth standards derived from Western populations (e.g., the Hadlock formula) to Chinese pregnancies may lead to over-diagnosis of FGR in constitutionally small but healthy fetuses. Therefore, the Chinese Expert Consensus on Fetal Growth Restriction (2019) recommends using NICHD Asian population-specific growth standards for accurate identification of FGR in this population (Buck Louis et al., 2015; Fetal Medicine Subgroup et al., 2022). Accordingly, and in alignment with relevant international guidelines, this study defines FGR as an EFW below the 10th percentile and severe FGR as an EFW below the third percentile (Fetal Medicine Subgroup et al., 2022; Lees et al., 2020; Shi et al., 2025; Society for Maternal-Fetal Medicine, 2020). For this analysis, cases were stratified into a non-severe group (EFW between the third and 10th percentiles) and a severe group (EFW below the third percentile). By systematically evaluating and comparing the diagnostic performance of ES in isolated non-severe versus severe FGR, this study aims to elucidate the impact of severity stratification on genetic diagnosis. The findings are expected to provide evidence for refining diagnostic pathways, enhancing the precision of genetic counseling, and informing pregnancy management decisions.

## 2 Materials and methods

### 2.1 Study population

This study included 164 singleton pregnancies diagnosed with isolated FGR by prenatal systematic ultrasound at our center between December 2022 and October 2025, without accompanying major structural anomalies, and who subsequently underwent invasive prenatal diagnostic procedures.

#### 2.1.1 Inclusion criteria

Gestational age was determined based on the last menstrual period or embryo transfer date and confirmed by first-trimester ultrasound. EFW was calculated from measurements of biparietal diameter, head circumference, abdominal circumference, and femur length, and assessed against Chinese (Asian) population fetal growth standards. Cases were stratified as non-severe FGR (EFW between the third and 10th percentiles for gestational age) or severe FGR (EFW below the third percentile). Follow-up systematic ultrasound examinations were performed every 2–4 weeks. Only singleton pregnancies with no major structural anomalies, or with isolated single or multiple ultrasound soft markers (e.g., absent nasal bone, echogenic intracardiac focus, pyelectasis), were included.

#### 2.1.2 Exclusion criteria

The exclusion criteria were: (1) positive maternal serum screening for congenital infections (i.e., *Toxoplasma gondii*, rubella virus, cytomegalovirus, and herpes simplex virus); (2) ultrasound detection of definitive fetal structural anomalies; and (3) multiple pregnancies.

### 2.2 Data collection

Maternal and fetal clinical data were obtained via electronic medical record review and telephone follow-up. Collected information included maternal age, obstetric history, mode of conception, gestational age at FGR diagnosis, pregnancy complications, detailed fetal biometry (EFW and its percentile), umbilical artery Doppler waveforms, and pregnancy outcomes.

The study protocol was approved by the Medical Ethics Committee of the People’s Hospital of Guangxi Zhuang Autonomous Region (Approval No. KY-KJT-2021-21). Written informed consent was obtained from all participants prior to any invasive procedures and genetic testing.

### 2.3 Genetic testing methods

#### 2.3.1 Chromosomal karyotyping and copy number variation sequencing

Following invasive sampling (amniocentesis or cordocentesis), G-banding chromosomal karyotype analysis was performed for all cases. Concurrently, fetal genomic DNA was extracted and subjected to genome-wide copy number variation screening using high-throughput sequencing-based copy number variation sequencing (CNV-Seq). This method systematically detects chromosomal deletions/duplications larger than 100 kb and aneuploidies.

#### 2.3.2 Trio exome sequencing

After obtaining comprehensive informed consent from the pregnant women and their spouses, trio-ES was performed for 36 cases from the non-severe FGR group and 89 cases from the severe FGR group (125 trios in total).

Exome capture was performed with the Roche KAPA HyperExome V2 kit. Captured libraries were sequenced on the BGI DNBSEQ-T7 platform using 150 bp paired-end reads, yielding >10 Gb of data to ensure >100 × average depth across target regions and >95% of targets with 20× coverage. Following quality control, clean reads were aligned to the GRCh37/hg19 human reference genome. Single nucleotide variants and small insertions/deletions were called using the GATK Best Practices pipeline and functionally annotated with Variant Effect Predictor. Common variants with a minor allele frequency >1% in gnomAD v2.1.1 were excluded. Exonic and splice site (±10 bp flanking exons) variants were retained, synonymous variants were filtered out unless predicted to be splice altering by SpliceAI. Phenotype-driven prioritization was conducted by intersecting variants with genes associated with fetal growth restriction and related ultrasonic soft markers, retrieved from OMIM, HPO, ClinGen, and DECIPHER. Rare variants were classified per the ACMG/AMP Standards and Guidelines for the Interpretation of Sequence Variants. Final pathogenicity assessments and clinical reporting decisions were reviewed and confirmed by a multidisciplinary team (MDT) including clinical geneticists, molecular geneticists, prenatal diagnosis specialists, and genetic counselors. P/LP variants were validated by Sanger sequencing in the proband and available family members.

### 2.4 Statistical analysis

Statistical analyses were conducted using SPSS software (version 25.0). Continuous variables are presented as mean ± standard deviation, and categorical variables as frequencies and percentages. Differences in clinical characteristics and the detection rates of P/LP variants via trio-ES between the non-severe and severe FGR groups were compared using the chi-square test. A two-sided *P* value <0.05 was considered statistically significant.

## 3 Results

### 3.1 Clinical characteristics

**FIGURE 1:**
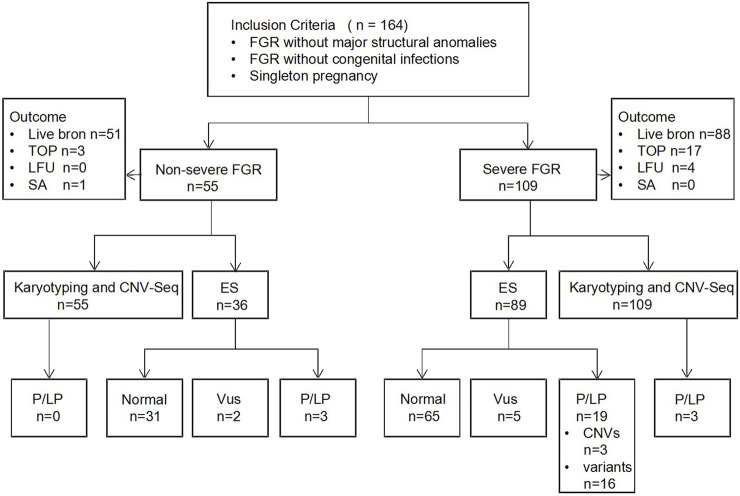
Flowchart of the cohort in isolated fetal growth restriction fetuses for Trio-ES analysis. LFU: lost to follow-up; TOP:termination of pregnancy; SA: Spontaneous Abortion; Trio-ES: Trio exome sequencing; VUS, variants of uncertain significance.

Our study enrolled a total of 164 cases with isolated FGR, as shown in Figure 1. Of these, 33.5% (55/164) were classified as non-severe FGR and 66.5% (109/164) as severe FGR. The two groups showed no significant differences in maternal age, gestational age at FGR diagnosis, parity (nulliparous vs. Multiparous), mode of conception (spontaneous vs. assisted), or history of adverse pregnancy outcomes. However, severe FGR was more frequently associated with preeclampsia (*P* = 0.02). While the presence of ultrasound soft markers did not differ significantly between groups, and abnormal umbilical artery Doppler indices (*P* = 0.04) were more common in the severe FGR group. The clinical characteristics are summarized in Table 1.

**TABLE 1:** Maternal and fetal characteristics.

Characteristic	Non-severe FGR mean ± SD/or n (%)	Severe FGR Mean ± SD/or n (%)	*P*-value
Maternal features			
Maternal age	31.2 ± 4.6 (years)	31.8 ± 4.9 (years)	0.40
Gestational age at diagnosis	26.5 ± 4.7(weeks)	27.4 ± 4 (weeks)	0.23
Nulipara	30/55 (54.5%)	64/109 (58.7%)	0.61
Abnormal reproductive history	10/55 (18.2%)	11/109 (10.1%)	0.14
Assisted reproduction	5/55 (9.1%)	6/109 (5.5%)	0.51
Gestational diabetes mellitus	7/55 (12.7%)	8/109 (7.3%)	0.26
Preeclampsia	1/55 (1.8%)	15/109 (13.8%)	0.02
Fetal features			
Male	34/55 (61.8%)	39/109 (35.8%)	<0.001
Ultrasound soft markers	11/55 (20.0%)	23/109 (21.1%)	0.87
Abnormal umbilical Doppler	2/55 (3.6%)	15/109 (13.8%)	0.04

### 3.2 Analysis of prenatal genetic testing results

All cases underwent karyotyping and CNV-seq. Trio-ES was performed on 65.5% (36/55) of the non-severe FGR group and 81.7% (89/109) of the severe FGR group. P/LP CNVs were detected in three fetuses in the severe FGR group, with none in the non-severe FGR group. In the non-severe FGR group, P/LP variants were identified in 8.33% (3/36) of fetuses, comprising two *de novo* autosomal dominant (AD) mutations and one compound heterozygous autosomal recessive (AR) variant. In the severe FGR group, P/LP variants were identified in 16 fetuses, with an overall detection rate of 18.0% (16/89). Among these positive cases, eight carried *de novo* mutations (six AD and two X-linked dominant), and eight had inherited variants. Of the 16 positive cases, 13 carried P/LP variants associated with short stature and/or intellectual disability. Additionally, three incidental findings unrelated to the FGR phenotype were detected. The difference in yield between our non-severe and severe FGR groups was not statistically significant (*P* = 0.08). Two variants of uncertain significance (VUS) were detected in the non-severe FGR group, and five VUS were identified in the severe FGR group. Detailed information on P/LP findings and VUS is provided in Tables 2–4, respectively.

**TABLE 2:** Comparison of trio-ES detection rates for P/LP findings among different subgroups.

Genetic findings	Non-severe FGR n (%)	Severe FGR n (%)	*P*-value
Trio-ES-tested	36/55 (65.5%)	89/109 (81.7%)	0.02
P/LP findings	3/36 (8.3%)	19/89 (21.3%)	0.08
P/LP CNVs	0	3/89 (3.3%)	​
P/LP variants	3/36 (8.3%)	16/89 (18.0%)	​
VUS variants	2/36 (5.6%)	5/89 (5.6%)	​
No P/LP variants identified	31/36 (86.1%)	65/89 (73.0)	​

**TABLE 3:** Pathogenic and likely pathogenic variants identified by trio-ES in fetuses with FGR.

Patient	Gene	Transcripts	Variant	Origin	Inheritance	ACMG classcification	Zygosity	Associated condition	Outcomes
Non-severe FGR									
1	BRD4	NM_001379291.1	c.1552-1G>A	*De novo*	AD	Pathogenic	Het	Cornelia de lange syndrome 6	TOP
2	GNAS	NM_000516.7	c.565_568del	*De novo*	AD	Pathogenic	Het	Pseudopseudohypoparathyroidism	TOP
	​	​	​	​	​	​	​	Pseudohypoparathyroidism Ia	​
	​	​	​	​	​	​	​	Pseudohypoparathyroidism Ic	​
3	CUL7	NM_014780.5	c.3129G>A	Pat	AR	Pathogenic	Het	3-M syndrome 1	TOP
	CUL7	NM_014780.5	c.4295-6_4313del	Mat		Pathogenic	Het		
Severe FGR									
4	FGFR3	NM_000142.5	c.1118A>G	*De novo*	AD	Pathogenic	Het	Achondroplasia	TOP
5	FGFR3	NM_000142.5	c.1138G>A	*De novo*	AD	Pathogenic	Het	Achondroplasia	TOP
6	COL1A1	NM_000088.4	c.1012G>A	Mat	AD	LP	Het	Osteogenesis Imperfecta	Live born
7	COL1A2	NM_000089.4	c.892G>A	Mat	AD	LP	Het	Osteogenesis Imperfecta	Live born
8	ASXL1	NM_015338.6	c.3416del	*De novo*	AD	LP	Het	Bohring-opitz syndrome	TOP
9	WDR45	NM_001029896.2	c.561_571del	*De novo*	XLD	LP	Het	Neurodegeneration with brain iron accumulation 5	TOP
10	GLI2	NM_005270.5	c.1189del	Pat	AD	LP	Het	Culler-jones syndrome	Live born
11	IGF1R	NM_000875.5	c.3349G>T	Pat	AD/AR	LP	Het	Insulin-like growth factor I, resistance to	Live born
12	CTCF	NM_006565.4	c.952 + 1G>T	*De novo*	AD	LP	Het	Mental retardation-21	TOP
13	CIC	NM_001386298.1	c.5395C>T	Mat	AD	LP	Het	Autosomal dominant intellectual developmental disorder	TOP
14	NFIX	NM_001271043.2	c.1182_1189del	*De novo*	AD	Pathogenic	Het	Malan syndrome marshall-smith syndrome	TOP
15	KMT2D	NM_003482.4	c.2758del	*De novo*	AD	Pathogenic	Het	Kabuki syndrome	TOP
16	CLCN4	NM_001830.4	c.592T>C	*De novo*	XLD	LP	Het	Raynaud-claes syndrome	Live born
17	GJB2	NM_004004.6	c.109G>A	Pat/Mat	AR	Pathogenic	Hom	Deafness, autosomal dominant 3A	Live born
18	BMPR2	NM_001204.7	c.1771C>T	Mat	AD	Pathogenic	Het	Familial pulmonary arterial hypertension	Live born
19	PKD1	NM_0010099 44.3	c.11177G>A	Pat	AD	Pathogenic	Het	Polycystic kidney disease 1	Live born

Pat, Parental inherited; Mat, Maternal inherited; AD, autosomal dominant; AR, autosomal recessive; XLD, X-linked dominant; LP, likely pathogenic; Het, Heterozygous; Hom, Homozygous; TOP, termination of pregnancy.

**TABLE 4:** VUS identified by trio-ES in Fetuses.

Patient	Gene	Transcripts	Variant	Origin	Inheritance	ACMG classcification	Zygosity	Associated condition	Outcomes
Non-severe FGR									
1	ABCC2	NM_000392.5	c.4348G>T	Pat	AR	VUS	Het	Dubin-johnson syndrome	Live born
	ABCC2	NM_000392.5	c.4239_4240dup	Mat	AR	Pathogenic	Het		
2	TUBG1	NM_001070.5	c.1144A>T	Mat	AD	VUS	Het	Complex cortical dysplasia with other brain malformations 4	Live born
Severe FGR									
3	GPNMB	NM_001005340.2	c.684T>A	Pat	AR	VUS	Het	Amyloidosis	Live born
	GPNMB	NM_001005340.2	c.565C>T	Mat	AR	Pathogenic	Het		Live born
4	ACADS	NM_000017.4	c.164C>T	Pat	AR	Pathogenic	Het	Short-chain acyl-CoA dehydrogenase deficiency	Live born
	ACADS	NM_000017.4	c.625G>A	Mat	AR	VUS	Het		Live born
5	KDM5C	NM_004187.5	c.4121T>G	Mat	XLR	VUS	Het	X-linked intellectual disability	Live born
6	ERCC4	NM_005236.3	c.2074C>T	Pat	AR	VUS	Het	Fanconi anemi complementation, group Q	TOP
	ERCC4	NM_005236.3	c.1787C>A	Mat	AR	VUS	Het		
7	DYNC2H1	NM_001080463.2	c.9834A>C	Pat	AR	VUS	Het	Short-rib thoracic dysplasia 3 with orwithout polydactyly	Live born

Pat, Parental inherited; Mat, Maternal inherited; AD, autosomal dominant; AR, autosomal recessive; XLR, X-linked recessive; VUS, variant of uncertain significance; Het, Heterozygous; TOP, termination of pregnancy.

### 3.3 Pregnancy outcomes

The live birth rate was 92.7% (51/55) in the non-severe FGR group and 80.7% (88/109) in the severe FGR group. Among these live-born neonates, gestational age at delivery did not differ significantly between the groups. However, those in the severe FGR group had significantly lower birth weight and shorter birth length.

For other pregnancy outcomes, the non-severe FGR group had 3 terminations of pregnancy, all following the detection of P/LP variants by trio-ES, and 1 miscarriage after amniocentesis. In contrast, the severe FGR group had 17 terminations of pregnancy. The indications for termination in this group were: 2 cases following P/LP CNV detection; 7 cases following the detection of P/LP variants by trio-ES; 1 case associated with a variant of VUS and severe phenotypic concerns; 4 cases due to severe preeclampsia; and 2 cases due to severe oligohydramnios. Detailed information on pregnancy outcomes is provided in Table 5.

**TABLE 5:** Pregnancy outcomes in different subgroups.

Pregnancy outcome	Non-severe FGR mean ± SD/or n (%)	Severe FGR Mean ± SD/or n (%)	*P*-value
Live born	51/55 (92.7%)	88/109 (80.7%)	0.11
Gestational age at birth	39.0 ± 1.6 (weeks)	38.1 ± 2.9 (weeks)	0.01
Brith weight	2876.4 ± 434.9 (g)	2444.3 ± 600.5 (g)	<0.001
Birth length	49.1 ± 2.6 (cm)	46.8 ± 3.6 (cm)	<0.001
Termination of pregnancy	3/55 (5.4%)	17/109 (15.6%)	0.06
P/LP CNVs	0	2/109 (1.8%)	​
P/LP variants	3/55 (5.4%)	8/109 (7.3%)	​
VUS Variants	0	1/109 (0.9%)	​
Severe Preeclampsia	0	4/109 (3.7%)	​
Severe oligohydramnios	0	2/109 (1.8%)	​
Spontaneous abortion	1/55 (1.8%)	0	​
Lost of follow-up	0	4/109 (3.7%)	​

## 4 Discussion

This study conducted a stratified analysis of 164 cases of isolated FGR, comparing maternal and fetal parameters, and evaluating the diagnostic yield of prenatal trio- ES between non-severe and severe strata. Our findings reveal that severe FGR is associated with a more complex pathophysiological profile. Critically, the cases demonstrate the clinical utility of ES even in the non-severe FGR population. This work underscores the etiological heterogeneity across the FGR severity spectrum, providing key insights for precise etiological investigation, risk stratification, and optimized clinical management.

All included cases underwent chromosomal karyotyping and CNV-Seq analysis. The detection rates for P/LP CNVs were 0.6% (1/164) by karyotyping and 1.8% (3/164) by CNV-Seq. All three CNV-positive cases belonged to the severe FGR group, involving a 15.81 Mb deletion at 10p15.3-p13, a 2.82 Mb duplication at 19q13.2, and a 1.84 Mb deletion at Xq23. Reported detection rates for P/LP CNVs in FGR cohorts from the literature are higher, ranging from 6.7% to 19% by karyotyping, with SNP-array providing an incremental yield of about 10% (Cai et al., 2022; Snijders et al., 1993). It is noteworthy, however, that these studies often included FGR cases with associated structural anomalies. For instance, one report noted that 96% of karyotype-positive FGR cases exhibited concurrent multisystem structural defects, with common findings including triploidy, trisomy 18, and trisomy 21. This suggests that the diagnostic utility of tests targeting P/LP CNVs is more limited in the context of isolated FGR.

In the non-severe FGR cohort, trio- ES was performed in 65. 5% of cases, compared with 81.7% in the severe FGR group, indicating that advanced genetic testing was more frequently utilized in cases of greater clinical severity. In China, ES is non-reimbursable under the national medical insurance scheme and remains a substantial financial burden for many families. This cost barrier constitutes the primary reason for its refusal. P/LP variants were identified in 8.3% of the non-severe FGR cases that underwent trio-ES. Case 1 carried a *de novo* heterozygous *BRD4* c.1552-1G>A variant, associated with Cornelia de Lange syndrome type 6 (OMIM 620568) (Garcia-Gutierrez and Garcia-Dominguez, 2021). Case 2 carried a *de novo* heterozygous *GNAS* c.565_568del variant. *GNAS* is an imprinted gene, variant on the paternal allele leads to Pseudopseudohypoparathyroidism (OMIM 612463), while variant on the maternal allele results in Pseudohypoparathyroidism Ia (OMIM 103580) or Pseudohypoparathyroidism Ic (OMIM 612462) (Juppner, 2021). Case 3 presented with compound heterozygous variants in *CUL7* (c.3129G>A and c.4295-6_4313del), inherited in trans, which are causative for 3-M syndrome type 1(OMIM: 273750) (Zhang et al., 2025). All three cases involved early-onset FGR attributable to variants in genes implicated in syndromic forms of growth retardation, conditions often associated with intellectual disability or endocrine/metabolic dysfunction.

Within the severe FGR group, P/LP variants were detected in 18.0% of cases that underwent trio-ES. Reduced long bone length is a frequent sonographic finding in FGR. Prior research indicates that among fetuses with isolated FGR characterized primarily by shortened long bones, the positive diagnostic rate by trio-ES can be as high as 11.9% (Shi et al., 2025). Consistently, four ES-positive cases in our severe FGR cohort presented with this feature. Cases 4 and 5 carried de novo heterozygous FGFR3 variants: c.1118A>G in Case 4 and c.1138G>A in Case 5, both responsible for Achondroplasia (OMIM 100800) (Zhang et al., 2024). Case 6 carried a heterozygous *COL1A1* c.1012G>A variant, and Case 7 carried a heterozygous *COL1A2* c.892G>A variant, both associated with Osteogenesis Imperfecta (OMIM 259420, OMIM166200) (Rodriguez Celin et al., 1993). Therefore, for FGR associated with shortened long bones, *FGFR3*, *COL1A1*, and *COL1A2* represent high-priority candidate genes. The resulting phenotypes predominantly involve disproportionate short stature, typically without cognitive impairment. A key distinction is that *FGFR3*-related achondroplasia demonstrates complete penetrance, whereas *COL1A1/COL1A2*-related Osteogenesis Imperfecta can exhibit incomplete penetrance and variable expressivity. This is illustrated by Cases 6 and 7, where the pathogenic variants were maternally inherited, yet both mothers had normal stature (1.55 m and 1.60 m) and no history of fractures or intellectual disability (Mei et al., 2022; Vajo et al., 2000).

The presence of microcephaly in conjunction with FGR is a significant clinical concern, often signaling a higher risk for adverse neurodevelopmental outcomes and potentially indicating an underlying genetic syndrome, thus warranting meticulous assessment (de Oliveira Junior et al., 2021; Haddad et al., 2024). In our severe FGR cohort, four cases with P/LP variants presented with reduced biparietal diameter and/or head circumference. Case 8 carried a *de novo* heterozygous *ASXL1* c.3416del variant linked to Bohring-Opitz Syndrome (OMIM 605039) (Russell et al., 1993). Case 9 carried a *de novo* mosaic *WDR45* c.561_571del variant (approximately 33% mosaicism) associated with Neurodegeneration with brain iron accumulation 5 (OMIM 300894) (Abe-Hatano et al., 2024). Case 10 carried a paternally inherited *GLI2* c.1189del variant related to Culler-Jones syndrome (OMIM: 615849) (Zhang et al., 2023; Yuan et al., 2025). Case 11 carried a paternally inherited *IGF1R* c.3349G>T variant causing resistance to Insulin-like growth factor I (OMIM: 270450) (Giabicani et al., 2020). With the exception of Case 10 (diagnosed at 34 weeks), these were early-onset cases. The implicated syndromes are primarily neurodevelopmental in nature, with genes such as ASXL1, WDR45, GLI2, and IGF1R playing crucial roles in embryonic development, cellular proliferation, and signaling pathways.

Several P/LP cases in the severe FGR group presented with symmetric FGR and were all early-onset. Case 12 carried a *de novo* heterozygous *CTCF* c.952 + 1G>T variant associated with Mental retardation-21 (OMIM 615502) (Wang et al., 2020). Case 13 carried a maternally inherited heterozygous *CIC* c.5395C>T variant linked to Intellectual developmental disorder, autosomal dominant 45 (OMIM: 617600) (Lu et al., 2017). Case 14 carried a *de novo NFIX* c.1182_1189 del variant associated with Malan syndrome (OMIM: 614753) and Marshall-Smith syndrome (OMIM: 602535) (Rai et al., 2018; Uzman et al., 2023). Case 15 carried a *de novo KMT2D* c.2758del variant causative of Branchial arch abnormalities syndrome (OMIM: 620186) and Kabuki syndrome 1 (OMIM: 147920) (Baldridge et al., 2020; Cuvertino et al., 2020). Case 16 had a *CLCN4* c.592T>C variant associated with Raynaud-Claes syndrome (OMIM: 300114) (Palmer et al., 1993). These genes are involved in transcriptional regulation, chromatin remodeling, and genome organization. Disorders associated with these genes typically feature significant neurological involvement (intellectual disability, developmental delay, brain anomalies), sometimes accompanied by skeletal or connective tissue abnormalities and multisystem effects.

Incidental findings, unrelated to the primary FGR phenotype, were identified in 3.4% (3/89) of severe FGR cases undergoing trio-ES. Case 17 carried a homozygous *GJB2* c.109G>A variant associated with autosomal recessive deafness (OMIM: 220290, OMIM:601544) (Meena and Ayub, 2017). Case 18 carried a *BMPR2* c.1771C>T variant causative of familial pulmonary arterial hypertension (OMIM: 178600) (Morrell et al., 2019). Case 19 carried a *PKD1* c.11177G>A variant linked to Polycystic kidney disease 1 (OMIM: 173900) (Lanktree et al., 2021).

For context, a study of 482 fetuses without structural anomalies on ultrasound reported an overall ES diagnostic yield of 1.24% (6/482), with 0.83% (4/482) having findings suggestive of moderate-to-severe disease (Daum et al., 2023). A larger investigation focusing on ultrasonographically normal fetuses found a trio-ES diagnostic rate of 1.42% (23/1766), of which 73.91% followed an autosomal dominant pattern and 40% were associated with a high risk for severe pathology (Wang et al., 2025). In our study, the ES diagnostic yield was 8.3% (3/36) in the non-severe FGR group (66.7% autosomal dominant) and 18.0% (16/89) in the severe FGR group (81.3% autosomal dominant). Both rates substantially exceed those reported for fetuses without structural anomalies. Additionally, postnatal follow-up was completed for all cases. One case with non-severe FGR, which did not undergo ES, received neonatal monogenic screening after delivery. A *de novo* pathogenic variant in the *PTPN11* c.1510C>G was detected and is associated with Noonan syndrome type 1 (OMIM: 163950) (Athota et al., 2020). For cases without ES, assessment relies only on current clinical phenotypes. This approach is insufficient because some children with monogenic disorders may show no obvious abnormalities in the neonatal or early infant period. Therefore, long-term follow-up remains essential.

The difference in yield between our non-severe and severe FGR groups was not statistically significant (*P* = 0.08), aligning with another report (Shi et al., 2025). However, it is noteworthy that in our study cohort, fewer cases in the non-severe FGR group underwent ES compared to the severe FGR group (n = 36 vs. n = 89). A similar disparity was observed in the study by Shi et al. (n = 29 vs. n = 106). This selection bias constitutes a major limitation of our study, likely attributable to the following factors: (1) Patient Decision-Making: The severity of FGR significantly influences the decision to pursue exome sequencing. Given the associated costs, parents of fetuses with severe FGR are more inclined to deem the financial investment justified for potentially clarifying the risk of birth defects. (2) Clinical Referral Patterns: In China, prenatal care is typically initiated in general obstetric departments, with complex cases referred to specialized prenatal diagnosis centers. As non-severe FGR is often under-prioritized by some obstetricians, primarily severe cases are referred. This results in a substantial proportion of non-severe FGR cases not receiving professional genetic counseling. Our center has also encountered several such cases where a genetic diagnosis was only achieved via ES after postnatal presentation of developmental delays.(3) Single-Center Design: As a single-center study, our sample size is limited. Future multi-center collaborations are needed to expand the cohort, particularly for non-severe FGR cases. In conclusion, we hope this study raises awareness of the clinical significance of non-severe FGR. Larger, prospective studies are warranted to provide robust evidence for genetic counseling and informed decision-making in such cases.

Although our findings did not indicate a statistically significant difference in the diagnostic yield of ES between severity-based strata of FGR, a stratified management approach remains clinically meaningful for the two groups. Regarding pregnancy outcomes, the rate of termination of pregnancy (TOP) was 5.4% in the non-severe FGR group. In all three TOP cases, trio-ES identified P/LP variants. This indicates that genetic abnormalities were the predominant factor driving termination of pregnancy decisions in this group, underscoring the clinical value of ES. In contrast, terminations of pregnancy in the severe FGR group were more frequent and resulted from diverse etiologies, including P/LP CNVs and variants, severe preeclampsia, oligohydramnios, and variants of VUS in the context of severe fetal phenotype. This pattern, combined with our data showing significantly higher incidences of maternal preeclampsia and abnormal umbilical artery Doppler studies in the severe FGR group, underscores that the management of severe FGR necessitates a dual focus on both maternal-placental health and fetal genetic factors. Furthermore, in the severe FGR group, decisions to decline ES may involve not only financial factors but also clinical perceptions. If a maternal-placental cause such as preeclampsia is identified, it may be perceived as a sufficient explanation, thereby obviating the need for genetic testing. Notably, severe FGR often appears to result from the confluence of multiple etiologies. For example, in Case 6, a *COL1A1* mutation was identified, yet the pregnancy was delivered preterm at 31 weeks due to concurrent severe maternal preeclampsia. Similarly, the case with the Xq23 deletion resulted in preterm delivery at 35 weeks secondary to preeclampsia. A multifactorial analytical approach is therefore crucial for comprehensive risk assessment during genetic counseling for severe FGR.

All cases with P/LP variants in the non-severe FGR group opted for TOP. In the severe FGR group, outcomes for P/LP cases were more varied: 50% chose TOP, while 50% continued the pregnancy. All P/LP variants in the latter group were inherited from a clinically unaffected parent (Cases 6, 7, 10, 11, 18, 19). Within this context, the clinical implications varied: some variants were associated with conditions exhibiting incomplete penetrance or variable expressivity (e.g., Osteogenesis Imperfecta, *GJB2*-related deafness). In one instance (Case 19), the variant was predictive of a late-onset disease (adult-onset polycystic kidney disease), inherited from a father who had subclinical imaging findings. Case 18 involved an X-linked dominant disorder in a female fetus. Following in-depth counseling, these pregnancies were continued, and the delivered infants have not exhibited related birth defects to date. This illustrates that a P/LP finding does not invariably lead to TOP, but rather that the decision is influenced by a composite of factors including disease severity (particularly neurological prognosis), age of onset, inheritance pattern, penetrance, and whether the variant is *de novo*.

While this study confirms the significant diagnostic value of ES in FGR, it also highlights a key practical challenge: the clinical counseling and management of variants of VUS. This is exemplified by one pregnancy in our cohort that was terminated following a VUS finding, a decision compounded by significant parental anxiety. This case underscores the profound dilemmas faced by families and clinicians. Disclosing VUS in prenatal testing inherently creates ethical tension between patient autonomy and the principle of non-maleficence. Consequently, rather than applying a uniform disclosure policy, we advocate for strengthened pre-test counseling. This process ensures that families understand the inherent uncertainty of VUS and can collaborate with clinicians to develop a personalized plan for result communication. Given the high genetic heterogeneity of FGR, ES provides the greatest diagnostic yield. Analysis should therefore be guided by the specific clinical phenotype, as replacing ES with targeted gene panel risks under-diagnosis. To manage these risks, ongoing genetic counseling both before and after testing is essential. Multidisciplinary team collaboration is a key to ensuring accurate interpretation and providing optimal family support.

## 5 Conclusion

In summary, this study demonstrates that stratifying isolated FGR according to NICHD Asian-based severity criteria effectively distinguishes groups with differing clinical and etiological profiles. Exome sequencing proves to be a valuable diagnostic tool not only in severe FGR but also in identifying consequential monogenic disorders within the non-severe FGR population. Its implementation, however, must be coupled with thoughtful strategies for managing uncertain results. Future research employing larger, prospective, and ideally multi-center cohorts is warranted to further clarify the cost-effectiveness of ES across the spectrum of isolated FGR, and to establish standardized frameworks for VUS counseling and clinical follow-up.

## Data Availability

The datasets presented in this study can be found in online repositories or in the supplementary material. The names of the repository and accession number can be found at: https://www.ncbi.nlm.nih.gov/clinvar/term=%22Medical%20Genetics%20and%20Prenatal%20Diagnosis%20Center%2C%20Guangxi%20Academy%20of%20Medical%20Sciences%20and%20the%20People%E2%80%99s%20Hospital%20of%20Guangxi%20Zhuang%20Autonomous%20Region%22%5bsubmitter%5d.
